# Nutritional Plasticity, Waste Bioconversion, and Insect Detoxification in the Anthropocene

**DOI:** 10.3390/insects16090915

**Published:** 2025-09-01

**Authors:** Anelise Christ-Ribeiro, Janaína Barreto Alves Zacheski, Andressa Jantzen da Silva Lucas, Larine Kupski

**Affiliations:** 1 Food and Chemistry School, Federal University of Rio Grande, Av. Itália, km 8, Rio Grande 96203-900, RS, Brazil; anelise.christ@hotmail.com (A.C.-R.); ninajbalves@gmail.com (J.B.A.Z.); 2 Institute of Technology, Federal University of Pará, R. Augusto Corrêa, no. 1, Belém 66075-110, PA, Brazil; andressajslucas@ufpa.br

**Keywords:** adaptation, ecological resilience, feed, food, gut microbiota

## Abstract

The Anthropocene, marked by rapid and extensive environmental changes, poses distinct evolutionary pressures and opportunities for species adaptation. Insects, among the most diverse and resilient taxa, exhibit notable dietary plasticity and the ability to convert low-value biomass—such as agro-industrial and urban waste—into usable nutrients. This review explores how these traits serve as adaptive strategies, enabling insects to thrive and expand into novel, human-altered habitats. We examine the evolution of insect nutritional requirements and how alternative diets influence physiological, behavioral, and reproductive traits, ultimately enhancing resilience to anthropogenic stressors. The capacity of insects to metabolize diverse substrates not only supports their role in food security and circular economy initiatives but also provides valuable insights into detoxification pathways and metabolic flexibility in environments rich in xenobiotics. By synthesizing key studies, we highlight the pivotal role insects play in redefining ecosystem functions under human influence. This review underscores the intersection of nutritional and evolutionary biology in understanding insect success in the Anthropocene, emphasizing the importance of nutritional knowledge for both ecological research and applied insect farming systems.

## Simple Summary

Insects are highly adaptable organisms that play an essential role in ecosystems. In the Anthropocene—a time of intense environmental changes driven by human activity—many insect species have shown a remarkable ability to survive by changing their diets and taking advantage of new food sources. This includes the ability to feed on agricultural and urban waste, turning low-value materials into nutrients that help them grow and reproduce. This review synthesizes insights from scientific literature, primarily focusing on advancements over the last decade, to explore how insect nutritional plasticity and detoxification mechanisms enable them to thrive in human-altered environments, bioconverting waste and supporting circular economy and food security. Their success also makes them valuable for sustainable food production and waste recycling. Understanding how insects process different types of food helps scientists not only improve insect farming practices but also better understand how living organisms respond to environmental challenges. This knowledge is important for both environmental sustainability and human food systems.

## 1. Introduction

The current geological era, informally designated as the Anthropocene, is characterized by human influence as the dominant transformative force in global terrestrial systems. These activities have resulted in a series of profound and accelerated environmental impacts, including severe climate change, massive habitat loss and fragmentation, and the widespread dissemination of pollutants across the planet [1]. Beyond these disruptive pressures, the Anthropocene is also characterized by the creation of new nutritional sources, which are shaped by intensive agriculture, urbanization, and, particularly, the large-scale generation of organic and inorganic waste. These by-products of human societies represent altered or entirely new resources that redefine the environments in which species must persist and evolve [2].

From the entire animal kingdom, insects emerge as a group of exceptional evolutionary success and diversity, colonizing practically all terrestrial and aquatic ecosystems [3]. Their remarkable biological plasticity—encompassing physiological, behavioral, and ecological flexibility—has been a fundamental driver of their persistence and proliferation across a vast range of environmental conditions over millions of years [4]. In the context of the Anthropocene, this inherent resilience is tested and redefined, as various insect species demonstrate an ability not only to survive but even to thrive and diversify in severely modified environments replete with new selective pressures [1,4].

One of the most notable manifestations of this plasticity in insects is their nutritional flexibility, which is the ability to utilize and thrive on a wide range of food sources, including those that are nutritionally challenging or contain complex compounds [2]. In the Anthropocene context, this characteristic assumes particular relevance in light of the growing availability of agro-industrial and urban waste. These by-products, once considered “trash,” emerge as abundant nutritional resources in anthropogenic environments, creating unique ecological niches [1]. The remarkable efficiency of insects in bioconverting this low-value biomass—transforming it into high-value protein and lipid biomass—should not be seen merely as a benefit for the circular economy and human food security [5]. On the contrary, this ability constitutes a crucial adaptive mechanism for their persistence, proliferation, and colonization of new habitats in the altered resource landscape of the Anthropocene, demonstrating a remarkable capacity for detoxification and metabolic assimilation of unconventional diets.

This review aims to deepen our understanding of how insect nutritional biology, particularly their capacity to metabolize and utilize waste-derived diets, serves as a fundamental pillar of their ecological adaptation and evolutionary success in the Anthropocene. It will explore the specific nutritional requirements of insects and how the plasticity of their physiological and metabolic response to dietary variations, including the utilization of complex substrates and sometimes xenobiotics, confers resilience upon them. The analysis will elucidate how these mechanisms of detoxification and utilization of altered resources contribute to the persistence of insect populations in a scenario of rapid environmental change, offering valuable insights not only for the optimization of sustainable production systems but, crucially, for a broader understanding of the dynamics of life on a planet dominated by human influence.

## 2. Nutritional Needs of Insects in the Anthropocene Context: Bases for Metabolic Plasticity

### 2.1. Essential Nutrients and Metabolic Plasticity of Insects in Anthropogenic Environments

Every living organism requires a set of nutritional compounds to sustain its development, reproduction, and maintenance of vital functions [6]. For insects, this premise is no different; however, the remarkable diversity of species and the wide range of habitats they colonize—including the significantly altered environments enriched by waste in the Anthropocene—reveal extraordinary nutritional and metabolic plasticity. This flexibility allows them not only to survive but also to thrive on diets with varied compositions and availabilities. The intrinsic ability of insects to assimilate nutrients from diverse and, at times, challenging substrates becomes a fundamental adaptive trait, especially given the emergence of new food sources from human activity [7]. Understanding these needs and how they are met in different dietary contexts is essential to unraveling the persistence strategies of insects on a transforming planet.

Macronutrients, such as carbohydrates, lipids, and proteins, constitute the essential pillars for insect development and maintenance [8]. Among these, proteins are crucial, acting as structural components, enzymatic catalysts, and regulatory elements of metabolic pathways. The remarkable ability of insects to convert low-biological-value proteins present in various organic wastes into high-value, protein-rich biomass [9] represents an ecological ‘upcycling’ strategy for nutrient utilization. In the Anthropocene, where the generation of proteinaceous waste is abundant, this ability not only offers sustainable solutions for waste management but also gives insects an adaptive competitive advantage, allowing them to exploit nutritional niches rich in protein sources that would be inaccessible to many other organisms [10]. The plasticity in utilizing different amino acid profiles in varied diets, often mediated by an adaptable gut microbiota, underscores the resilience of insects in the face of variability in available protein sources in anthropogenic environments.

Lipids play essential multifunctional roles in insect physiology, acting as constituents of cell membranes, hormonal precursors, essential energy reserves, and water conservation barriers in the cuticle [8]. A distinctive and adaptively relevant characteristic is the inability of insects to synthesize sterols (such as cholesterol), making them strictly dependent on dietary sources [11]. In the Anthropocene context, the abundance of agro-industrial and urban waste, which frequently contains phytosterols (in plant materials such as bran) or cholesterol (in animal by-products like tallow), represents a unique nutritional opportunity. The ability of various insect species to efficiently extract and metabolize these lipids from complex and varied sources—a nutritional challenge for many other organisms—is a crucial adaptation that allows them to successfully colonize and exploit new, abundant food niches in environments altered by human activity [12]. The fatty acid composition in the insect, as discussed later, directly reflects the lipid composition of the diet, indicating remarkable plasticity in nutrient incorporation for optimizing their biological functions.

Carbohydrates, primarily in the form of monosaccharides and disaccharides, are the primary energy source for insects, in addition to being essential for the formation of chitin, the main component of the exoskeleton [8]. The proliferation of waste in the Anthropocene, particularly that rich in complex polysaccharides such as cellulose, hemicellulose, starch, and pectin (originating from agricultural crops, food scraps, etc.), represents a vast energy repository [12]. The ability of particular insect species to hydrolyze these complex and recalcitrant chains into simpler sugars is a crucial metabolic adaptation. This capacity is often mediated not by a robust endogenous enzymatic arsenal (which is rarer in insects), but by a symbiotic and diversified gut microbiota, which provides the necessary enzymes for the breakdown of these polysaccharides [13,14]. This nutritional symbiosis allows insects to capitalize on and thrive in energy sources that would be inaccessible to many other organisms, demonstrating an adaptive strategy for exploiting niches dominated by anthropogenic by-products.

In addition to macronutrients, minerals are inorganic compounds that insects cannot synthesize and, therefore, are essential for a myriad of biological functions, including the formation of skeletal structures, maintenance of ionic balance, and acting as cofactors in critical enzymatic systems [8,15]. In the Anthropocene scenario, where mineral landscapes can be significantly altered by intensive agricultural practices, pollution, and the generation of waste with unconventional mineral profiles, the ability of insects to acquire and utilize these elements becomes evidence of their adaptability [16]. The literature demonstrates that insects such as *Tenebrio molitor* can increase their dry matter and mineral composition in response to diets enriched with calcium (Ca) [17], a vital mineral for molecular bonding in invertebrate structures. This flexibility in extracting essential minerals from diverse sources, such as phosphorus (P) or potassium (K) from bran and calcium (Ca) from eggshells, present in organic waste, is a strategy that allows these organisms to thrive in environments where natural mineral availability can be unpredictable or suboptimal, contributing to their ecological resilience.

Vitamins are essential organic compounds that, like minerals, cannot be synthesized by most insects, requiring their intake through the diet to act as cofactors in numerous metabolic reactions and growth processes [8]. In Anthropocene environments, where food sources may present variable and even deficient vitamin profiles, especially in diets based solely on homogeneous waste, the ability of insects to adapt to diverse diets becomes a significant adaptive advantage. This allows them to extract the necessary spectrum of vitamins from a wide range of substrates, compensating for specific deficiencies through the breadth of their diet [18]. It is notable that, in many cases, the symbiotic gut microbiota of insects plays a crucial role in the synthesis and availability of specific vitamins, such as those in the B complex, thereby further strengthening the host’s nutritional resilience in the face of the uncertainties of the anthropogenic environment [13]. The complexity and potential for nutrient bioaccumulation in insects are evidenced by their nutritional composition, as detailed in comparison with the requirements of other organisms in Table 1.

### 2.2. The Crucial Role of Gut Microbiota in Insect Adaptation in the Anthropocene

The interaction between the host insect and its gut microbiota is a crucial and dynamic component that profoundly influences the insect’s physiology, behavior, and adaptive capacity [21]. Far from being merely commensal, the microbiota represents a crucial co-evolutionary mechanism that confers invaluable adaptive advantages to insects, especially in the exploration of unconventional diets and persistence in challenging Anthropocene environments [13]. This microbial symbiosis is fundamental for the following:

- Improved Digestive Efficiency: The microbiota enables the insect to digest and assimilate nutrients from complex and recalcitrant substrates, such as cellulose, hemicellulose, and lignin, often abundant in agro-industrial and urban waste [14]. This capacity broadens the spectrum of resources that the insect can use as food, transforming what would be useless to others into a viable energy source
- Synthesis of Essential Nutrients: In nutritionally unbalanced or deficient diets, the microbiota can synthesize essential vitamins (notably B-complex) and amino acids, filling nutritional gaps for the host and ensuring full development [8].
- Detoxification Mechanisms and Xenobiotic Tolerance: The gut microbiota plays a critical role in the biotransformation and detoxification of plant secondary compounds, as well as xenobiotics and pollutants present in waste and the anthropogenic environment. This ability to neutralize potentially toxic substances is a cornerstone of insect resilience in contaminated ecosystems [22].
- Immunity Modulation and Pathogen Resistance: A balanced microbiota contributes to the overall health of the insect, aiding in defense against pathogens and strengthening its immune response [23].

The composition and function of insect gut microbiota are highly plastic, actively responding to variations in diet and environment [21].

This adaptive plasticity of the holobiont (insect and its associated microorganisms) enables insects like the black soldier fly, *Tenebrio molitor* larvae, to thrive on organic waste-based diets, conferring a significant competitive advantage in colonizing and exploiting emerging nutritional niches in the Anthropocene. Concrete examples of this plasticity include the following:

- *Hermetia illucens* (Black Soldier Fly Larvae): Their gut microbiota demonstrates remarkable shifts in community structure and function when larvae are reared on diverse organic waste streams (e.g., municipal food waste, agricultural residues). These microbial adaptations enhance the breakdown of complex biopolymers and optimize nutrient cycling, allowing for efficient bioconversion and biomass accumulation [22].
- *Tenebrio molitor* (Mealworm Larvae): When fed diets containing recalcitrant materials like polystyrene, the mealworm’s gut microbiome adapts, with an increased abundance of specific bacterial taxa that contribute to polymer depolymerization and utilization, showcasing its role in novel waste valorization [24].
- Detoxification Mechanisms: The gut microbiota also plays a crucial role in detoxifying xenobiotics. For instance, in the coffee berry borer (Hypothenemus hampei), gut bacteria facilitate the detoxification of caffeine, enabling the insect to thrive on coffee beans as its primary food source [25].
- Nutrient Compensation: In various insect species, particularly those on nutritionally imbalanced or deficient diets, changes in the gut microbial community can lead to enhanced synthesis of essential vitamins (e.g., B-complex vitamins) and amino acids, thus compensating for dietary gaps and ensuring host development [8,13].

## 3. Dietary Plasticity and Persistence Strategies in the Anthropocene: Evidence and Implications

Given the established nutritional needs and the complexity of the Anthropocene food landscape, insects have developed remarkable strategies to optimize nutrient acquisition and utilization [26]. The exploitation of alternative diets and the efficiency in waste conversion are not mere optimizations in production systems but represent a direct manifestation of the nutritional and adaptive plasticity of these species in the face of the abundance of unconventional resources generated by human activity [2]. The observed variation in the nutritional composition of insects in response to the diet provided to them serves as compelling evidence of their metabolic flexibility and the ability to directly integrate and transform environmental resources, reflecting the dynamics of new anthropogenic landscapes.

### Metabolic Flexibility and Exploitation of Anthropogenic Substrates

Historically, the search for insect nutrient sources has focused on more economical and accessible alternatives [12]. However, from an evolutionary and ecological perspective, the continuous search for cheaper and more reliable sources can be recontextualized as the adaptive capacity of insects to capitalize on abundant and predictable resources that emerge in anthropogenic landscapes [2]. Agro-industrial and urban waste, such as cereals and brewery by-products, represent vast reservoirs of nutrients that, while of low economic value for humans, offer a rich ecological niche for insect species with metabolic plasticity [27]. The technique of cultivating microorganisms on substrates like grains, aiming to increase protein content, can be seen as a human intervention that mimics or accelerates microbial bioconversion processes already occurring in nature, where insects and their microbiotas act in the degradation of complex biomass [14]. Even the use of pest-infested grains can be interpreted as a way for insects to exploit previously compromised resources, indicating a resource optimization strategy in environments with an abundance of defective or residual materials.

The body composition of insects is a direct reflection of their diet and developmental environment, revealing remarkable phenotypic plasticity that enables them to modulate their constitution in response to nutritional availability. It is observed that, while the protein fraction of specific insect species tends to be relatively stable, regardless of the substrate on which they are reared—indicating a homeostatic prioritization of essential amino acid and protein composition for biological functionality [28]—the lipid fraction (and its fatty acid profile) demonstrates significant variation, directly reflecting the lipid composition of the diet. This flexibility in the assimilation and incorporation of lipids is a crucial adaptation for exploiting waste-derived diets, which often exhibit diverse and fluctuating lipid profiles. By adjusting their fat accumulation and composition, insects can optimize their energy and structural reserves based on the most abundant and accessible resources in the anthropogenic environment [29]. Additionally, the ability to incorporate other compounds such as minerals and vitamins from the diet into their biomass underscores the extreme interconnection between environmental resources and insect constitution, demonstrating how life can thrive and optimize itself in scenarios of unconventional resources [30].

Table 2 synthesizes various studies investigating the influence of the nutritional composition of alternative diets on insects, offering empirical examples of physiological plasticity and, in some cases, adaptive responses to varied dietary environments. These studies, when re-evaluated from the perspective of the Anthropocene, illustrate the strategies by which insects can persist and thrive in a world characterized by altered food sources and dynamic selective pressures.

Plasticity in growth and development is a fundamental adaptive strategy for insects in unpredictable environments, such as those created by the Anthropocene. Studies with *Drosophila melanogaster* (fruit fly) demonstrate that macronutrient restriction during the larval stage has a more deleterious impact on adult size than caloric restriction, evidencing physiological trade-offs in scenarios of undernutrition. However, the observation that a rich adult diet can mitigate the adverse effects of larval malnutrition highlights a recovery and resilience mechanism [26] crucial for species facing fluctuations in food quality and availability in anthropogenic niches (such as urban environments or agricultural waste). Analogously, compensatory growth in *Bombyx mori* (silkworm) [32], where individuals overcome developmental delays after periods of nutritional restriction, illustrates an adaptation to optimize fitness under variable resource conditions. This ability to ‘recover’ is vital for reproductive success and population persistence in environments where food supply can be intermittent, a common characteristic of waste sources in the Anthropocene [37].

Metabolic and biochemical adaptations enable insects to optimize nutrient utilization and cope with dietary challenges in anthropogenic environments. In *Spodoptera frugiperda* (fall armyworm), the variation in the gut metabolome profile in response to larval diet demonstrates remarkable metabolic plasticity, where specific biochemical pathways are activated to optimize the digestion and assimilation of different food sources [7,31]. This capacity is crucial for exploiting heterogeneous diets found in waste. Similarly, studies with *Sesamia cretica* Lederer (pink stem borer) reveal that the nutritional adequacy of cultivars is intrinsically linked to the presence of protein inhibitors, plant compounds that act as antinutritional factors by interfering with protein digestion in insect guts, and the plant’s biochemical properties [34,38]. These plant-derived compounds, such as Kunitz and Bowman–Birk type inhibitors targeting insect trypsin and chymotrypsin, or cystatins inhibiting cysteine proteases, are key antinutritional factors that disrupt protein digestion in insect guts. Although the focus is agricultural, this research illustrates the selective pressure for the evolution of detoxification mechanisms and tolerance to secondary compounds present in food sources. In response to such dietary challenges, insects have developed various counter-adaptations, including the overexpression of specific digestive proteases, reliance on symbiotic microbiota to detoxify inhibitors, or the exploitation of pre-processing methods (e.g., fermentation, thermal treatment) that can reduce inhibitor activity. The ability to circumvent these chemical defenses, whether through endogenous enzymes or with the aid of the gut microbiota, represents a form of ecotoxicological adaptation that enables insect persistence in environments where the chemical quality of resources may be compromised or presents challenges.

The allocation of nutritional resources for immune function represents a crucial adaptive strategy for insects exposed to an increasing spectrum of pathogens and environmental stressors in the Anthropocene. A study with the cricket *Gryllodes sigillatus* showed that immunity in males and females was significantly higher on protein-rich diets and that populations exhibited evidence of evolution in response to dietary manipulation over generations [35]. This illustrates how macronutrient availability can shape immunological resilience and, by extension, the survival capacity of a population in environments with greater pathogenic pressure or toxicity. The ability to efficiently allocate nutrients for defense, especially proteins essential for the immune response, confers an adaptive advantage in scenarios of increasing biotic and abiotic challenges, highlighting the role of diet in modulating insect fitness.

Reproductive adaptation and resource choice are crucial for species persistence in dynamic Anthropocene environments. In *Psyttalia concolor* Szépligeti, the variation in longevity and parasitism capacity in response to different carbohydrate sources, including sugars and honeydew [33], demonstrates plasticity in the utilization of energy resources to optimize reproductive fitness. This flexibility is vital for species that may encounter varied or intermittent food sources in environments altered by human activity. Similarly, for the glassy-winged sharpshooter (*Homalodisca vitripennis*), fecundity is primarily determined by the quality of the adult diet [36]. The ability to optimize oogenesis and egg production on different plant species (or mixtures), in addition to a preference for specific sources, illustrates behavioral and physiological foraging and resource allocation strategies in complex landscapes. These adaptations allow insects to maximize their reproductive potential by exploiting the most efficient nutritional sources available, whether in monocultures, urban environments, or vegetable waste, ensuring species continuity.

## 4. Detoxification Mechanisms and Biochemical Adaptation to Anthropogenic Substrate

The remarkable ability of insects to colonize and thrive in emerging nutritional niches in the Anthropocene, particularly those rich in agro-industrial and urban waste, demands more than simple flexibility in macronutrient and micronutrient acquisition [28]. These substrates, although abundant, can present significant challenges, such as the presence of recalcitrant complex polysaccharides, plant secondary metabolites (antinutritional or toxic compounds), and xenobiotics of anthropogenic origin (like pesticides and industrial contaminants) [6]. These substrates, although abundant, can present significant challenges, such as the presence of recalcitrant complex polysaccharides, plant secondary metabolites (including antinutritional or toxic compounds), and xenobiotics of anthropogenic origin (e.g., pesticides and industrial contaminants) [39]. These pathways represent an essential adaptive line of defense, allowing the biotransformation of potentially harmful compounds into less toxic and more easily excretable forms, underscoring their resilience and success in a chemically ever-changing environment.

**Figure 1:**
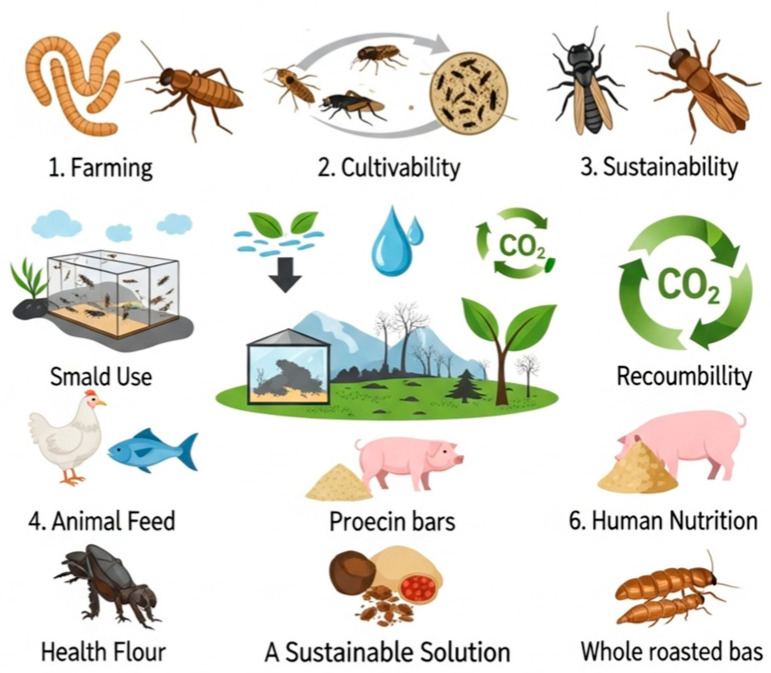
Detoxification and biochemical adaptation mechanisms of insects in anthropocene environments.

Detoxification mechanisms (Figure 1) in insects are orchestrated by a diverse set of enzymatic systems that act in two main phases of biotransformation. Phase I involves the introduction or exposure of polar groups to lipophilic molecules, thereby increasing their reactivity. Cytochrome P450 monooxygenases (P450s) are the most abundant and versatile enzymes in this phase, catalyzing oxidation, reduction, and hydroxylation reactions of a vast range of substrates, including pesticides, plant secondary metabolites, and other xenobiotics. Phase II detoxification involves the conjugation of modified molecules from Phase I with hydrophilic endogenous compounds, increasing their solubility for excretion. The primary Phase II enzymes include glutathione S-transferases (GSTs) and carboxylesterases (CarEs), which also play a role in ester hydrolysis and insecticide resistance. The expression and activity of these enzymes are highly plastic and can be induced by the presence of certain compounds in the diet or environment. This enzymatic plasticity is fundamental for the adaptation of insects to varied and complex food sources found in anthropogenic environments, allowing them to neutralize antinutritional or toxic compounds present in waste and thereby optimize nutrient assimilation [21,40,41].

The insect gut microbiota, already highlighted for its contribution to the digestion of complex carbohydrates and the synthesis of essential nutrients [14,21], plays an equally, if not more, vital role in the detoxification of undesirable compounds in anthropogenic environments. This symbiotic partnership is a robust adaptation that expands the host’s metabolic capabilities beyond its own genome [13]. Symbiotic microorganisms can directly degrade toxins, biotransform xenobiotics (such as residual pesticides in food or environmental pollutants) into less harmful forms, and even release nutrients sequestered by antinutritional compounds. For example, gut bacteria can hydrolyze cyanogenic glycosides or oxalates, which would be toxic to the host insect. However, it is crucial to acknowledge that microbial biotransformations are not invariably benign. While less studied in insect systems compared to detoxification pathways, the potential for microbial toxification exists, where nominally non-toxic precursors are transformed into more toxic secondary metabolites that could be absorbed by the host or excreted in the frass [22]. Candidate pathways for such toxification include the deconjugation of glycosides to release reactive aglycones (e.g., free cyanide from cyanogenic glycosides), the formation of nitrosation products from nitrates, or the partial oxidation of complex hydrocarbons and chemical additives present in certain waste streams. While direct evidence of significant adverse effects from such microbial toxification pathways in insect farming is still scarce, and the primary role of the gut microbiota remains detoxification, this area represents a crucial data gap requiring further investigation to fully understand the safety and metabolic fate of diverse substrates in bioconversion systems [42]. Furthermore, the microbiota contributes to maintaining the integrity of the intestinal barrier, thereby protecting the insect from the absorption of harmful substances [25]. The ability of the microbiota’s composition to rapidly adapt to changes in diet and xenobiotic exposure—thereby shaping the detoxification “arsenal” available to the insect—is evidence of the holobiont’s adaptive plasticity. This interdependence between the insect and its microbial symbionts confers a crucial competitive advantage, allowing insect species to thrive and exploit food sources that would be unsustainable without this metabolic partnership [38].

## 5. Challenges and Risk Management in the Application of Insects in the Circular Bioeconomy

While the potential of insects in waste bioconversion and their contribution to a circular bioeconomy are undeniable and promising, it is crucial to address the complexities and potential risks associated with these applications. A balanced assessment requires considering the limitations and challenges that may arise in practice, especially regarding product safety and environmental impact.

Despite the remarkable nutritional plasticity and robust detoxification mechanisms of insects, mediated by enzymatic systems such as cytochrome P450 monooxygenases, glutathione S-transferases, and carboxylesterases, waste biotransformation by insects does not guarantee the complete elimination of all contaminants. Studies have consistently shown that, depending on the feeding substrate composition, undesirable substances can be transferred and accumulated in insect biomass. This includes, but is not limited to, heavy metals (e.g., cadmium and lead), persistent organic pollutants (such as per- and polyfluoroalkyl substances—PFAS), pesticides, and veterinary drug residues [43,44] This bioaccumulation is a significant concern for food safety, especially when insects or their derivatives are intended for human or animal consumption. Furthermore, a considerable portion of these contaminants can be preferentially partitioned into insect excrements, known as frass. With the growing commercialization of frass as an agricultural fertilizer, there is a latent risk of reintroducing these contaminants into the food web and the environment, necessitating rigorous monitoring and clear guidelines for frass management [45,46].

Concurrently, the ability of certain insects, such as mealworm larvae, to interact with plastic materials has generated significant interest, often interpreted as “plastic degradation.” However, it is essential to differentiate between ingestion and partial depolymerization from complete mineralization. Many studies indicate that, instead of complete biodegradation of the polymer into harmless compounds like CO_2_ and water, what often occurs is the fragmentation of plastic into smaller microplastics or the assimilation of chemical additives present in the original material [24]. This distinction is crucial, as the persistence of microplastics and the possible release of additives into insect biomass or the environment can raise new ecological and safety concerns, requiring further research on the ultimate fate of these materials and their potential impacts.

Finally, microbiological safety is a paramount consideration in the bioconversion of organic waste by insects. Although the insect gut microbiota plays a crucial role in modulating immunity and protecting against pathogens, the effectiveness in reducing antimicrobial pathogens during insect rearing processes is highly dependent on operational conditions and the sanitary quality of raw materials. The survival of public health pathogens, such as *Salmonella* spp. and pathogenic *Escherichia coli*, and the persistence of antimicrobial resistance genes (ARGs) have been documented under certain insect rearing conditions and with specific raw materials [44,47]. These findings underscore the imperative need to implement rigorous Good Manufacturing Practices (GMPs) and quality control systems in insect production for food and feed. The validation of processes that ensure the inactivation or significant reduction of pathogenic microorganisms and the minimization of antimicrobial resistance dissemination is essential to ensure the safety and sustainability of this promising industry.

## 6. Conclusions

In the Anthropocene, insects exemplify remarkable adaptability through their nutritional plasticity and sophisticated detoxification mechanisms, often facilitated by a crucial gut microbiota. These capabilities enable them to thrive in human-altered environments and efficiently bioconvert diverse, often challenging, substrates into valuable biomass.

Beyond optimizing production systems, insects represent a sustainable and innovative source of novel proteins and organic compounds, critical for global food and feed security with a reduced environmental footprint. Their role extends significantly to climate initiatives, acting as efficient decomposers of industrial and agricultural waste, which mitigates landfill burden and greenhouse gas emissions. Furthermore, the potential for certain species in carbon sequestration through biomass conversion warrants deeper investigation.

Crucially, the complex insect–microbiome interaction positions insects as a robust biological model for fundamental research. This symbiosis offers an unparalleled opportunity to unravel intrinsic evolutionary and adaptive mechanisms in response to diverse environmental conditions. Employing advanced techniques like metabolomics and metatranscriptomics in these systems will profoundly enhance our understanding of microbial influence on host physiology, bioconversion capabilities, and overall resilience.

Ultimately, a deeper comprehension of insect nutritional biology and detoxification mechanisms is essential, not only for optimizing sustainable production but also for leveraging insects as pivotal ecological agents and scientific models in addressing the pressing challenges of the Anthropocene.

## Figures and Tables

**Table 1:** Nutritional requirements of different species of animals.

Insect Composition	Protein	Crude Fiber	Carb	Fats	Vitamin A	Vitamin B2	Vitamin C	Fe	Ca	Zn	P	Mg
	(%)				(mg/100 g)							
Cirina forda (larvae)	20.2	1.8	ND *	ND *	3.0	2.2	2.0	64.0	15.4	8.6	110.0	1.9
Brachytrupes membranaceus (adult)	53.4	15.0	15.1	53.0	0.0	0.0	0.0	0.7	9.2	ND *	126.9	0.1
Brachytrupes spp. (adult)	6.3	1.0	ND *	ND *	0.0	0.0	0.0	0.7	9.2	ND *	126.9	0.1
Rhynchophorus phoenicis (larvae)	28.4	2.8	ND *	ND *	11.3	2.2	4.3	12.2	39.6	ND *	126.4	7.5
Carebara vidua (adult)	42.5	9.1	ND *	38.2	12.4	3.2	10.3	25.2	15.4	ND *	125.5	5.2
Humans’ total requirements of nutrients	Protein	Crude Fiber	Carb	Fats	Vitamin A	Vitamin B2	Vitamin C	Fe	Ca	Zn	P	Mg
	RDA ** (g/day)	AI ***	RDA ** (g)		RDA (μg)	RDA ** (mg)			AI ***	RDA ** (mg)		
Women (age 19–30)	46.0	25.0	130.0	ND *	700.0	1.1	75.0	18.0	1000.0	8.0	700.0	400.0
Women (age 31–50)	46.0	21.0	130.0	ND *	700.0	1.1	75.0	18.0	1000.0	8.0	700.0	420.0
Men (age 19–30)	56.0	38.0	130.0	ND *	900.0	1.3	90.0	8.0	1000.0	11.0	700.0	400.0
Men (age 31–50)	56.0	30.0	130.0	ND *	900.0	1.3	90.0	8.0	1000.0	11.0	700.0	420.0
Animals’ total requirements of nutrients	Protein	Crude Fiber	Carb	Fats	Vitamin A	Vitamin B2	Vitamin C	Fe	Ca	Zn	P	Mg
	(%)				IU	mg	mg/ kg		(%)	mg/ kg	(%)	
Swine (7–11 kg)	ND *	ND *	ND *	ND *	2200.0	3.5	* ND	100.0	0.8	100.0	0.65	0.04
Swine (75–100 kg)	ND *	ND *	ND *	ND *	1300.0	2.0	* ND	40.0	0.52	50.0	0.47	0.04
Tilapia	41.3	ND *	ND *	ND *	4769.0	ND *	600.0	60.0	ND *	79.51	0.65	* ND
Deer	ND *	ND *	ND *	ND *	ND *	ND *	ND *	50.0	0.35	50.0	0.25	0.2
Chickens (6–8 weeks)	19–21	ND *	ND *	ND *	1500.0	3.0	ND *	80.0	0.8	40.0	0.3	ND *

* ND: Not determined; ** RDA: Recommended Dietary Allowance; *** AI: Adequate Intake; Source: adapted from Refs. [6,7,10,11,19,20].

**Table 2:** Influence of the nutritional composition of alternative diets on insects.

Source	Insect	Stage	Parameters	Results	Conclusion	References
Varied protein and carbohydrate content (macronutrient restriction) and caloric density (calorie restriction)	*Drosophila melanogaster* (fruit fly)	Larval and adult	Body weight, wing and femur size	Macronutrient restriction was more detrimental to adult size than caloric restriction. For adult body weight, a rich adult diet mitigated the negative effects of larval malnutrition for both types of diets. Poor diet of larvae caused smaller wing and femur sizes without recovery using adult diet.	The nutritional conditions of the larvae play a dominant role in determining adult body weight and wing and femur size, the adult diet may adjust body weight as flies age.	[26]
Artificial diet based on corn, rice, or cotton leaves	*Spodoptera frugiperda* (fall armyworm)	Eclosion to the sixth instar	Identify differences in metabolite profiles of the larval gut	The metabolome of the midgut of insects varied according to the larval diet	Effects of diet on the metabolome, differential digestive metabolism, and identified marker metabolites.	[31]
Mulberry leaves, artificial diet, and artificial diet + mulberry leaves	*Bombyx mori* (silkworm)	1st instar to the 3rd instars, 4th instar to mature larvae (cocooning stage)	Physiological characteristics and the underlying mechanisms	Compensatory growth occurred, and genes related to metabolism and development in the midgut of the silkworm showed differences.	Compensated for developmental delay and body weight loss after changing nutritional status, as well as severe physiological changes (body weight gain, altered digestive juice activity, and altered gene expression in the midgut).	[32]
Sugars (glucose, fructose, sucrose, trehalose, melibiose, melezitose, and sorbitol); two types of honeydew	*Psyttalia concolor* Szépligeti	-	Longevity and parasitism capacity	The longevity of females increased with sorbitol and melibiose and males with hexose nectars. Feeding with honeydew showed better results.	Source of carbohydrates (sugar or honeydew) is important for longevity and reproduction, especially for females.	[33]
Diets with protein to carbohydrate ratio and nutritional content	Cricket (*Gryllodes sigillatus*)	Replicate populations (for >37 generations)	Hemocyte counts, the zone of inhibition, and total phenoloxidase	After three generations, in males and females, immunity was higher on protein. Although females exhibited superior immunity for all assays, the sexes showed similar immune changes across diets.	Indicated that populations evolved with dietary manipulation.	[34,35]
Six sugarcane cultivars (total phenolic, flavonoids, and anthocyanins contents)	Pink stem stalk borer (*Sesamia cretica* Lederer)	Fifth instar larvae	Nutritional responses and body weight; digestive enzymatic activity	One cultivar was less suitable due to low relative consumption rates and relative growth rate, related to low nutritional level, biochemical properties, and high concentration of protein inhibitors.	Indicated remarkable differences in the nutritional properties and digestive function; significant variations in the phytochemical metabolites were detected	[34]
Cowpea, sunflower, sorghum, and a mixture of the three plant species	Glassy-winged sharpshooter (*Homalodisca vitripennis* (Germar)	9 wk oviposition period and adults emerging	Nymphal development, oogenesis, and fecundity	On nymphal diets, active females produced more eggs in plant mixtures. In choice testes, adult females opted for cowpea, but most eggs were deposited in sorghum.	Fecundity is largely determined by the quality of the adult diet, although the stimulus that initiates oogenesis does not appear to be related to nutrition.	[36]

## Data Availability

Data are available on request from the corresponding authors.
